# Supplementary material for: Differential Expression of Proteins in an Atypical Presentation of Autoimmune Lymphoproliferative Syndrome
Source: Int J Mol Sci. 2022 May 11;23(10):5366. doi: 10.3390/ijms23105366 (PMC9140392; doi:10.3390/ijms23105366)
Supplement: Supplementary file 1 [file ijms-23-05366-s001.zip › ijms-1692249-supplementary/Supplementary Material/~WRL1284.tmp]

**Supplementary Table 1.** Up-Regulated and down-Regulated Proteins detected with CV ≤ 0.15, two peptides per protein (one unique) and ANOVA *p* ≤ 0.05.

| **UP** | | | | | |
| --- | --- | --- | --- | --- | --- |
| Protein | Accession | Gene | Fold change | Log_2_ Fold change | Functions |
| DNA mismatch repair protein Mlh3 | Q9UHC1 | MLH3 | 42.44138 | 5.4074 | DNA mismatch repair protein; DNA binding protein. Implicated in maintaining genomic integrity during DNA replication and after meiotic recombination |
| Pericentrin | O95613 | PCNT | 26.88151 | 4.748542 | Interacts with the microtubule nucleation component gamma-tubulin and is likely important to normal functioning of the centrosomes, cytoskeleton, and cell-cycle progression |
| HLA class I histocompatibility antigen, A-3 alpha chain | Q5SRN5 | HLA-A | 22.10183 | 4.466094 | A class I molecule expressed in nearly all cells that play a central role in the immune system by presenting peptides derived from the endoplasmic reticulum lumen |
| Plastin-2 | P13796 | LCP1 | 20.20757 | 4.336824 | Actin binding protein; Plays a role in the activation of T-cells in response to costimulation through TCR/CD3 and CD2 or CD28. Modulates the cell surface expression of IL2RA/CD25 and CD69 |
| Cytokine receptor-like factor 3 | Q8IUI8 | CRLF3 | 14.13389 | 3.821086 | DNA binding protein nucleotide phosphatase; may negatively regulate cell cycle progression at the G0/G1 phase |
| Cat eye syndrome critical region protein 2 | Q9BXF3 | CECR2 | 13.03693 | 3.704533 |  |
| Nebulin (Fragment) | H0Y786 | NEB | 12.11385 | 3.598585 | Component of the cytoskeletal matrix |
| Vimentin | P08670 | VIM | 11.71188 | 3.549901 | Type III intermediate filament protein responsible along with microtubules and actin microfilaments for maintaining cell shape and integrity of the cytoplasm and stabilizing cytoskeletal interactions. Also, Vim functions as an organizer of several other critical proteins involved in cell attachment, migration and signaling |
| Rho guanine nucleotide exchange factor 12 | Q9NZN5 | ARHGEF12 | 10.96599 | 3.454965 |  |
| Heterogeneous nuclear ribonucleoprotein K | P61978 | NRNPK | 10.06337 | 3.331042 |  |
| Isoform 2 of Dedicator of cytokinesis protein 4 | Q8N1I0-2 | DOCK4 | 9.190393 | 3.200127 |  |
| Apolipoprotein C-III | B0YIW2 | APOC3 | 7.904548 | 2.982683 |  |
| Uncharacterized protein C4orf50 | E9PNW5 | C4orf50 | 7.646219 | 2.934746 | Uncharacterized protein |
| PWWP domain-containing protein 2B | Q6NUJ5 | PWWP2B | 7.241632 | 2.856315 |  |
| Origin recognition complex subunit 2 | Q13416 | ORC2 | 6.791745 | 2.763782 | Replication origin binding protein. Part of the essential complex for initiation of DNA replication |
| Dedicator of cytokinesis protein 11 | Q5JSL3 | DOCK11 | 6.712385 | 2.746826 | Guanyl-nucleotide exchange factor; dedicator of cytokinesis 11 |
| Platelet basic protein | P02775 | PPBP | 6.553452 | 2.712255 |  |
| Replication protein A 70 kDa DNA-binding subunit | P27694 | RPA1 | 6.387384 | 2.675225 | DNA-directed RNA polymerase nucleotidyltransferase;  binds and stabilizes single stranded DNA intermediates that form during DNA replication or upon DNA stress; it prevents their re-annealing and recruits and activates different proteins and complexes involved in DNA metabolism |
| Semaphorin-6D | Q8NFY4 | SEMA6D | 6.191382 | 2.630261 | Membrane bound-signaling molecule |
| Platelet factor 4 | P02776 | PF4 | 6.035233 | 2.59341 |  |
| Protein S100-A8 | P05109 | S100A8 | 5.128781 | 2.358616 |  |
| Lymphocyte-specific protein 1 | P33241 | LSP1 | 5.058883 | 2.338819 |  |
| Coatomer subunit beta' | P35606 | COPB2 | 4.978586 | 2.315736 |  |
| Prelamin-A/C | Q5TCI8 | LMNA | 4.969452 | 2.313087 | Can accelerate smooth muscle cell senescence. It acts to disrupt mitosis and induce DNA damage in vascular smooth muscle cells, leading to mitotic failure, genomic instability, and premature senescence. |
| NACHT, LRR and PYD domains-containing protein 14 | Q86W24 | NLRP14 | 4.761412 | 2.25139 |  |
| Rho GDP-dissociation inhibitor 2 | P52566 | ARHGDIB | 4.694546 | 2.230986 | G-protein modulator signaling molecule  Regulator of the cytoskeleton |
| Nck-associated protein 5 | A0A0A0MS79 | NCKAP5 | 4.566907 | 2.191217 |  |
| E3 ubiquitin-protein ligase HECTD1 | Q9ULT8 | HECTD1 | 4.552745 | 2.186737 | Directly transfers the ubiquitin to targeted substrates |
| Glucosidase 2 subunit beta | P14314 | PRKCSH | 4.532818 | 2.180408 | N-linked glycan-processing enzyme |
| Platelet factor 4 variant | P10720 | PF4V1 | 4.359601 | 2.124196 | Chemokine |
| Coactosin-like protein | Q14019 | COTL1 | 4.237172 | 2.083102 | Actin-binding protein |
| Nascent polypeptide-associated complex subunit alpha, muscle-specific form | E9PAV3 | NACA | 4.22137 | 2.077711 | Basic helix-loop-helix transcription factor |
| Axonemal dynein light chain domain-containing protein 1 | Q5T1B0 | AXDND1 | 4.047582 | 2.01706 | Axonemal dynein lightchain domain-containing protein 1 |
| Glucose-6-phosphate 1-dehydrogenase | P11413 | G6PD | 3.925691 | 1.972947 |  |
| Dynein heavy chain 6, axonemal | Q9C0G6 | DNAH6 | 3.783173 | 1.919597 | Microtubule binding motor protein |
| Serine--tRNA ligase, cytoplasmic | P49591 | SARS | 3.755766 | 1.909107 | RNA binding protein aminoacyl-tRNA synthetase |
| Heat shock protein HSP 90-beta | P08238 | HSP90AB1 | 3.698296 | 1.886861 | Chaperone involved in signal transduction, protein folding and degradation and morphological evolution; is thought to play a role in gastric apoptosis and inflammation |
| Immunoglobulin heavy constant gamma 2 | P01859 | IGHG2 | 3.615016 | 1.854002 |  |
| Cytoplasmic protein NCK2 | O43639 | NCK2 | 3.576822 | 1.838678 | Involved in cytoskeletal reorganization |
| Histone H2B type 1-D | P58876 | HIST1H2BD | 3.565417 | 1.834071 | Component of the nucleosomes which, in addition to wrap and compact DNA into chromatin, play a central role in transcription regulation, DNA repair, DNA replication and chromosome stability |
| Isoform 2 of Dystonin | Q03001-8 | DST | 3.406367 | 1.768234 |  |
| Coronin-1A | P31146 | CORO1A | 3.37597 | 1.755302 | Non-motor actin binding proteins  Involved in a variety of cellular processes, including cell cycle progression, signal transduction, apoptosis, and gene regulation |
| Heterogeneous nuclear ribonucleoprotein Q | O60506 | SYNCRIP | 3.3363 | 1.738249 |  |
| Isoform 2 of Vasoactive intestinal polypeptide receptor 2 | P41587-2 | VIPR2 | 3.229025 | 1.691099 |  |
| Filamin-C | Q14315 | FLNC | 3.207691 | 1.681535 |  |
| Xin actin-binding repeat-containing protein 2 | A4UGR9 | XIRP2 | 3.191186 | 1.674093 |  |
| Lipopolysaccharide-responsive and beige-like anchor protein | P50851 | LRBA | 3.189025 | 1.673115 |  |
| Hemoglobin subunit gamma-2 | P69892 | HBG2 | 3.098604 | 1.631618 |  |
| Histone H2A type 1-H | Q96KK5 | HIST1H2AH | 3.073629 | 1.619943 |  |
| Hemoglobin subunit delta | P02042 | HBD | 3.05859 | 1.612867 |  |
| Cofilin-1 | P23528 | CFL1 | 2.978019 | 1.574353 | Regulator of the cytoskeleton |
| Isoform 2 of Heat shock protein HSP 90-alpha | P07900-2 | HSP90AA1 | 2.974058 | 1.572433 | Involved in cell cycle control and signal transduction |
| HCG1745306, isoform CRA_a | G3V1N2 | HBA2 | 2.895402 | 1.533764 |  |
| Isoform 4 of Xin actin-binding repeat-containing protein 2 | A4UGR9-4 | XIRP2 | 2.877506 | 1.524819 |  |
| Transforming protein RhoA | P61586 | RHOA | 2.815206 | 1.49324 | Associated with cytoskeleton organization. Overexpression of this gene is associated with tumorcell proliferation and metastasis. |
| Hemoglobin subunit alpha | P69905 | HBA1 | 2.811146 | 1.491158 | Human alpha globin |
| 6-phosphogluconate dehydrogenase, decarboxylating | P52209 | PGD | 2.77722 | 1.473642 |  |
| Peroxiredoxin-1 (Fragment) | A0A0A0MSI0 | PRDX1 | 2.758974 | 1.464132 |  |
| Synaptotagmin-like protein 2 | A0A0U1RR07 | SYTL2 | 2.732614 | 1.450282 | Plays a role in RAB27A-dependent vesicle trafficking and controls melanosome distribution in the cell periphery |
| Isoform 4 of Platelet glycoprotein 4 | P16671-4 | CD36 | 2.718027 | 1.44256 |  |
| Isoform 5 of Serine/threonine-protein kinase tousled-like 1 | Q9UKI8-5 | TLK1 | 2.71272 | 1.43974 | Serine/threonine kinase that may be involved in the regulation of chromatin assembly |
| Phosphoglycerate kinase 1 | P00558 | PGK1 | 2.701341 | 1.433676 | Cofactor for polymerase alpha |
| G patch domain-containing protein 4 | A0A0A0MRK1 | GPATCH4 | 2.657459 | 1.410047 |  |
| Versican core protein | P13611 | VCAN | 2.60464 | 1.381084 |  |
| Actin-related protein 2/3 complex subunit 3 | O15145 | ARPC3 | 2.556426 | 1.354128 |  |
| Tyrosine-protein kinase ITK/TSK | Q08881 | ITK | 2.551291 | 1.351227 |  |
| Ubiquitin-40S ribosomal protein S27a (Fragment) | J3QTR3 | RPS27A | 2.550911 | 1.351013 |  |
| Isoform 3 of Ubiquitin carboxyl-terminal hydrolase 7 | Q93009-3 | USP7 | 2.517224 | 1.331834 | Deubiquitinates target proteins such as p53 between others, promoting proteasomal degradation |
| Thrombospondin-1 | P07996 | THBS1 | 2.483254 | 1.312232 | Adhesive glycoprotein that mediates cell-to-cell and cell-to-matrix interactions. |
| Hemoglobin subunit beta | P68871 | HBB | 2.482989 | 1.312078 | Hemoglobin subunit beta |
| ATP-binding cassette sub-family A member 1 | O95477 | ABCA1 | 2.461312 | 1.299428 |  |
| Protein unc-13 homolog D | Q70J99 | UNC13D | 2.411428 | 1.269888 | Appears to play a role in vesicle maturation during exocytosis and is involved in regulation of cytolytic granules secretion |
| Calreticulin | P27797 | CALR | 2.394849 | 1.259935 | Multifunctional protein that acts as a major Ca(2+)-binding (storage) protein |
| Na(+)/H(+) exchange regulatory cofactor NHE-RF1 | O14745 | SLC9A3R1 | 2.366835 | 1.242959 |  |
| Coiled-coil domain-containing protein 39 | Q9UFE4 | CCDC39 | 2.360073 | 1.238831 | Motility of cilia and flagella |
| Prohibitin | P35232 | PHB | 2.345002 | 1.229589 | Is proposed to play a role in human cellular senescence and tumor suppression. Antiproliferative activity is reported to be localized to the 3' UTR, which is proposed to function as a trans-acting regulatory RNA |
| Calpastatin | P20810 | CAST | 2.342036 | 1.227763 |  |
|  |  |  |  |  |  |
| **DOWN** | | | | | |
| Cytosolic carboxypeptidase 2 | Q5U5Z8 | AGBL2 | 0.402469 | -1.31305 | G-protein metalloprotease |
| Cysteine and glycine-rich protein 1 | P21291 | CSRP1 | 0.400022 | -1.32185 |  |
| Thrombospondin-2 | P35442 | THBS2 | 0.399148 | -1.32501 |  |
| Serotransferrin | P02787 | TF | 0.386377 | -1.37192 | Receptor serine protease transfer/carrier protein; transports iron from the intestine, reticuloendothelial system, and liver parenchymal cells to all proliferating cells in the body |
| Kinesin-like protein KIF18A | Q8NI77 | KIF18A | 0.36719 | -1.4454 | Microtubule binding motor protein; kinesin |
| Isoform 3 of Polycystic kidney disease 2-like 1 protein | Q9P0L9-3 | PKD2L1 | 0.360671 | -1.47124 |  |
| Kinesin-like protein KIF22 | Q14807 | KIF22 | 0.346076 | -1.53084 | Involved in spindle formation and the movements of chromosomes during mitosis and meiosis. |
| Rho-associated protein kinase 1 | Q13464 | ROCK1 | 0.343188 | -1.54293 |  |
| Isoform PKP3b of Plakophilin-3 | Q9Y446-2 | PKP3 | 0.34304 | -1.54355 | Participates in linking cadherins to intermediate filaments in the cytoskeleton |
| 2,4-dienoyl-CoA reductase, mitochondrial | Q16698 | DECR1 | 0.342193 | -1.54712 |  |
| Myosin regulatory light chain 12A | P19105 | MYL12A | 0.341905 | -1.54833 | Involved in DNA damage repair by sequestering the transcriptional regulator apoptosis-antagonizing transcription factor (AATF)/Che-1 which functions as a repressor of p53-driven apoptosis |
| Xaa-Pro dipeptidase | P12955 | PEPD | 0.333076 | -1.58608 | Metalloprotease; nucleic acid binding transcription factor; recycling of proline |
| Protein FAM47A | A0A0C4DGW7 | FAM47A | 0.326965 | -1.61279 |  |
| Fructose-bisphosphate aldolase C | P09972 | ALDOC | 0.297765 | -1.74776 |  |
| Crk-like protein | P46109 | CRKL | 0.290316 | -1.78431 |  |
| ERC protein 2 (Fragment) | H7C4G9 | ERC2 | 0.28808 | -1.79546 | G-protein modulator; membrane traffic protein; regulator of neurotransmitter release |
| Zinc finger protein 492 | Q9P255 | ZNF492 | 0.282596 | -1.82318 |  |
| Band 3 anion transport protein | P02730 | SLC4A1 | 0.262393 | -1.9302 | Transporter; anion exchanger in the erythrocyte plasma membrane |
| PDZ and LIM domain protein 7 | Q9NR12 | PDLIM7 | 0.254756 | -1.97281 |  |
| Eukaryotic translation initiation factor 4 gamma 2 | P78344 | EIF4G2 | 0.244238 | -2.03364 | Plays a role in the switch from cap-dependent to IRES-mediated translation during mitosis, apoptosis and viral infection. Cleaved by some caspases and viral proteases. |
| Ankyrin-3 | Q12955 | ANK3 | 0.232868 | -2.10242 | Links the integral membrane proteins to the underlying spectrin-actin cytoskeleton and plays key roles in activities such as cell motility, activation, proliferation, contact, and the maintenance of specialized membrane domains. |
| Unconventional myosin-XVIIIa | Q92614 | MYO18A | 0.220753 | -2.17949 |  |
| Citrate synthase, mitochondrial (Fragment) | H0YH82 | CS | 0.208337 | -2.26301 | Krebs tricarboxylic acid cycle enzyme |
| Integrin alpha-M | P11215 | ITGAM | 0.150349 | -2.73361 | Integral membrane protein |
| UTP--glucose-1-phosphate uridylyltransferase | Q16851 | UGP2 | 0.144282 | -2.79304 |  |
| Guanine nucleotide-binding protein G(z) subunit alpha | P19086 | GNAZ | 0.142569 | -2.81026 |  |
| Isoform 2 of Protocadherin gamma-B6 | Q9Y5F9-2 | PCDHGB6 | 0.140918 | -2.82707 |  |
| D-aminoacyl-tRNA deacylase 1 | Q8TEA8 | DTD1 | 0.136309 | -2.87505 |  |
| Ras GTPase-activating protein 3 | Q14644 | RASA3 | 0.129194 | -2.95239 |  |
| Isoform 2 of Pleckstrin homology domain-containing family O member 2 | Q8TD55-2 | PLEKHO2 | 0.115905 | -3.10899 | Part of the Innate immune system |
| Cardiomyopathy-associated protein 5 | Q8N3K9 | CMYA5 | 0.099686 | -3.32646 |  |
| Myosin-11 | P35749 | MYH11 | 0.085022 | -3.55602 |  |
| Spectrin beta chain, erythrocytic | P11277 | SPTB | 0.048564 | -4.36397 | Cell membrane organization and stability |
| Transcription factor E2F7 | Q96AV8 | E2F7 | 0.039747 | -4.65302 | Regulation of cell cycle progression |
