# Supplementary material for: Differential Expression of Proteins in an Atypical Presentation of Autoimmune Lymphoproliferative Syndrome
Source: Int J Mol Sci. 2022 May 11;23(10):5366. doi: 10.3390/ijms23105366 (PMC9140392; doi:10.3390/ijms23105366)
Supplement: Supplementary file 1 [file ijms-23-05366-s001.zip › ijms-1692249-supplementary/Supplementary Material/Supplementary Figures.pdf]

Differential expression of proteins in an atypical presentation of Autoimmune Lymphoproliferative Syndrome

Dulce Ma. Delgadillo[1], Adriana Ivonne Céspedes-Cruz[2], Emmanuel Ríos-Castro[1], Ma. Guadalupe Rodríguez Maldonado[2], Mariel López-Nogueda[2], Miguel Angel Márquez-Gutierrez[2], Lorena Rámirez-Reyes[1], Rocío Villalobos-Manzo[3], Misael Domínguez-Fuentes[1] and José Tapia-Ramírez*[1,3]

1 Unidad de Genómica, Proteómica y Metabolómica. Laboratorio Nacional de Servicios Experimentales (LaNSE) Centro de Investigación y de Estudios Avanzados, Ciudad de México, México. cdelgadi-llo@cinvestav.mx; erios@cinvestav.mx; lramirez@cinvestav.mx;

2 Unidad Médica de Alta Especialidad (UMAE) Centro Médico Nacional La Raza Hospital General.

3 Departamento de Genética y Biología Molecular, Centro de Investigación y de Estudios Avanzados, Ciudad de México, México. jtapia@cinvestav.mx; rvillalobos@cinvestav.mx

***Correspondence: Departamento de Genética y Biología Molecular, Centro de Investigación y de Estudios Avanzados, Ciudad de México, México. jtapia@cinvestav.mx**

Supplementary Figures

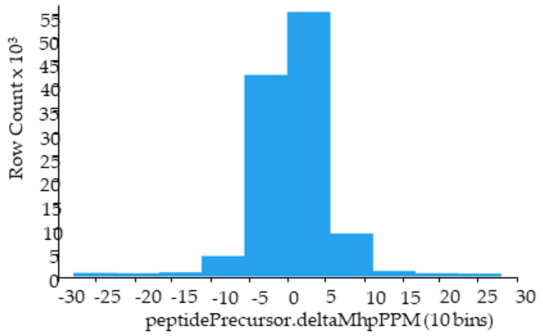

(**a**)

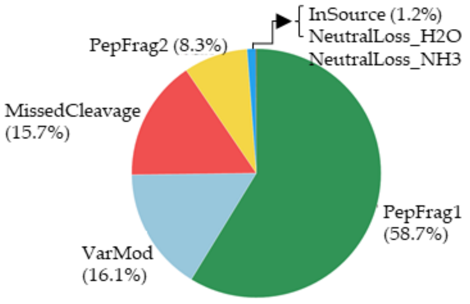

(**b**)

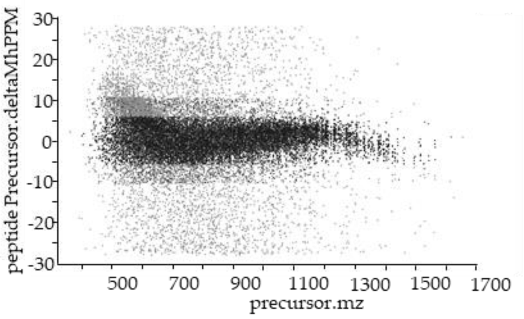

(**c**)

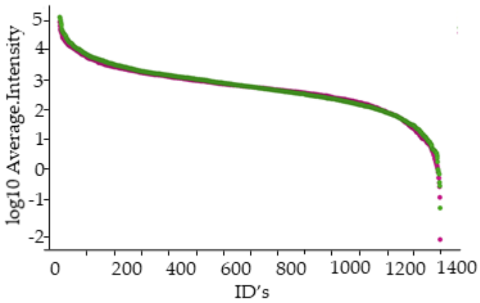

(**d**)

Supplementary Figure S1. Peptide/protein reliability and confidence. a, Histogram representing a total of 116081 detected peptides (z=2^{+} or higher); 82.08% of them fall into an error maximum of ± 5 ppm, this result indicates that Synapt G2-S*i* mass spectrometer was properly calibrated throughout the MS analysis. b, Pie chart showing the types of peptides identified by Progenesis software. 58.7% "PepFrag1", peptides of high quality because they satisfy a theoretical model contained in the search algorithm, which considers 14 physicochemical parameters such as, a complete enzymatic digestion, a high number of ion products matched, a good correlation of the sum of product ion intensities between the intensity of precursor ions among others (Li et al, 2009). These peptides span most of the pie chart which is desirable; also, 16.1% peptides were considered as "VarMod" which are peptides that contain some modification including PTMs; 15.7% were classified as "MissedCleavage", indicating efficiency in trypsin digestion. Like Pepfrag1, VarMod" and "MissedCleavage" peptides are identified with high quality, however, they are searched during the database search pass 2. Further, 8.3% were considered as "PepFrag2", they are peptides identified without restrictions on product ion intensity. Additionally, peptides that presented "neutral loss" of H_2O and NH_3 as well as "InSource" ions (peptides fragmented in the ion source) summed up 1.2 % of total peptides. "PepFrag2", "NeutralLoss" and "InSource" peptides have less reliability, so it is desired that they cover a fraction of the pie chart, which is consistent with our results (9.5% of total). c, PepFrag1 peptides are concentrated at a maximum of ± 10 ppm throughout the analyzed *m*/*z* range. d, Dynamic range of quantified proteins. In magenta control and in green ALPS-U sample. "*y*" axis, average of Hi3 intensities for each detected protein (values are represented as base 10 logarithm). "*x*" axis, number of quantified proteins (IDS). Quantified proteins comprise around 6 orders of magnitude, which probe sensibility of Synapt G2-S*i*; beside this, both ranges have a precise fit each other, demonstrating that it was injected a similar number of peptides for both conditions during the normalization in MS^{E} mode, so that both samples were comparable in this study.

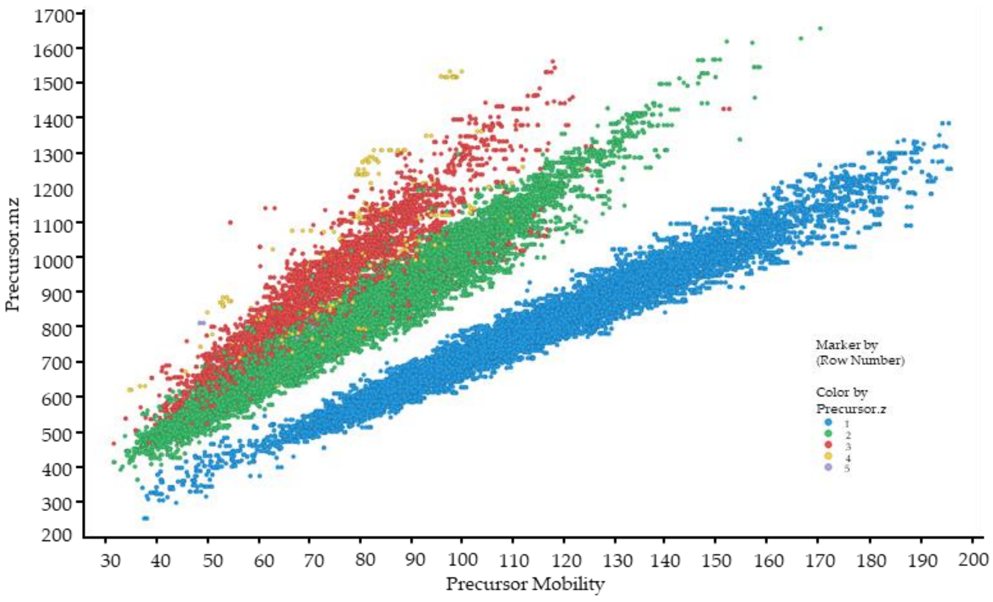

(a)

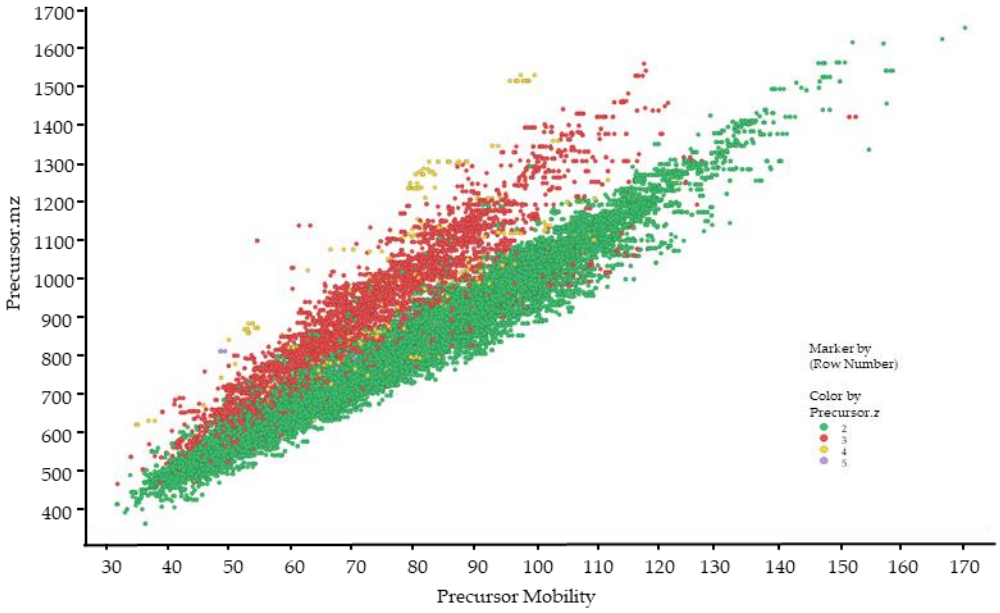

(b)

Supplementary Figure S2. Ion-mobility spectrometry (IM-MS). The use of ion mobility (IM-MS) in mode HDMS^E allows those ions within the mobility cell have distinct kinetic energies influenced under the physicochemical characteristics of the molecules as well as mobility cell conditions. In this way, ions are grouped into charge state-dependent clusters (Ríos-Castro et al 2020); in this manner, is possible to obtain the Drift Times of the ions detected with correspondent $[M + 2H]^{2+}$ (or higher) that are related to peptide signals in a *.rul file, which is used in a spectrometric method in UDMS^E mode in order to apply *quasi*-specific collision energy to each peptide detected in the mass spectrometer, which improves the quality of spectra led to leading to an increase in coverage and identifications. a) 185460 ions were detected using HDMS^E mode; blue dots correspond to the ions z=1$^+$; green dots, ions z=2$^+$; red dots, ions z=3$^+$; yellow dots, ions z=4$^+$ and purple dots, ions z=5$^+$. b) Ions with z=1+, were discarded in the final analysis (UDMS^E mode), in consequence 116081 peptides were used to identify and quantify.

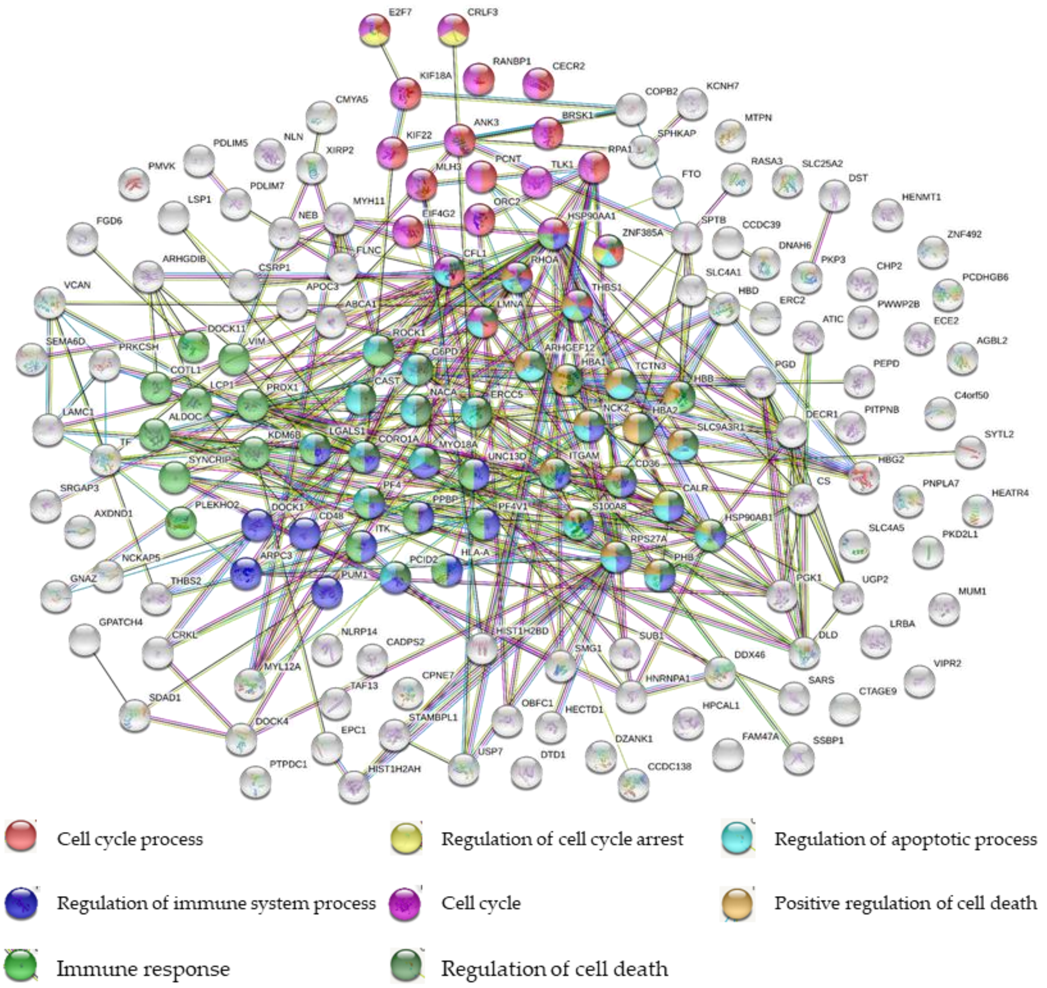

Supplementary Figure S3. Network generated by the String database analysis. Interaction network of all identified proteins, up-regulated and down-regulated. Colors show the functional classification of the proteins in the network as indicated.
