# Supplementary material for: Differential Expression of Proteins in an Atypical Presentation of Autoimmune Lymphoproliferative Syndrome
Source: Int J Mol Sci. 2022 May 11;23(10):5366. doi: 10.3390/ijms23105366 (PMC9140392; doi:10.3390/ijms23105366)
Supplement: Supplementary file 1 [file ijms-23-05366-s001.zip › ijms-1692249-supplementary/Supplementary Material/Supplementary Table S1.pdf]

Supplementary Table S1. Up-Regulated and down-Regulated gene expression for proteins detected with CV ≤ 0.15, two peptides per protein (one unique) and ANOVA $p \leq 0.05$.

UP					
Protein	Accession	Gene	Fold change	Log_2 Fold change	Functions
DNA mismatch repair protein Mlh3	Q9UHC1	MLH3	42.44138	5.4074	DNA mismatch repair protein; DNA binding protein. Implicated in maintaining genomic integrity during DNA replication and after meiotic recombination
Pericentrin	O95613	PCNT	26.88151	4.748542	Interacts with the microtubule nucleation component gamma-tubulin and is likely important to normal functioning of the centrosomes, cytoskeleton, and cell-cycle progression
HLA class I histocompatibility antigen, A-3 alpha chain	Q5SRN5	HLA-A	22.10183	4.466094	A class I molecule expressed in nearly all cells that play a central role in the immune system by presenting peptides derived from the endoplasmic reticulum lumen
Plastin-2	P13796	LCP1	20.20757	4.336824	Actin binding protein; Plays a role in the activation of T-cells in response to costimulation through TCR/CD3 and CD2 or CD28. Modulates the cell surface expression of IL2RA/CD25 and CD69
Cytokine receptor-like factor 3	Q8IUI8	CRLF3	14.13389	3.821086	DNA binding protein nucleotide phosphatase; may negatively regulate cell cycle progression at the G0/G1 phase
Cat eye syndrome critical region protein 2	Q9BXF3	CECR2	13.03693	3.704533	

Nebulin (Fragment)	H0Y786	NEB	12.11385	3.598585	Component of the cytoskeletal matrix
Vimentin	P08670	VIM	11.71188	3.549901	Type III intermediate filament protein responsible along with microtubules and actin microfilaments for maintaining cell shape and integrity of the cytoplasm and stabilizing cytoskeletal interactions. Also, Vim functions as an organizer of several other critical proteins involved in cell attachment, migration and signaling
Rho guanine nucleotide exchange factor 12	Q9NZN5	ARHGEF12	10.96599	3.454965	
Heterogeneous nuclear ribonucleoprotein K	P61978	NRNPK	10.06337	3.331042	
Isoform 2 of Dedicator of cytokinesis protein 4	Q8N1I0-2	DOCK4	9.190393	3.200127	
Apolipoprotein C-III	B0YIW2	APOC3	7.904548	2.982683	
Uncharacterized protein C4orf50	E9PNW5	C4orf50	7.646219	2.934746	Uncharacterized protein
PWWP domain-containing protein 2B	Q6NUJ5	PWWP2B	7.241632	2.856315	
Origin recognition complex subunit 2	Q13416	ORC2	6.791745	2.763782	Replication origin binding protein. Part of the essential complex for initiation of DNA replication
Dedicator of cytokinesis protein 11	Q5JSL3	DOCK11	6.712385	2.746826	Guanyl-nucleotide exchange factor; dedicator of cytokinesis 11
Platelet basic protein	P02775	PPBP	6.553452	2.712255	
Replication protein A 70 kDa DNA-binding subunit	P27694	RPA1	6.387384	2.675225	DNA-directed RNA polymerase nucleotidyltransferase; binds and stabilizes single stranded DNA intermediates that form during DNA replication or upon DNA stress; it

					prevents their re-annealing and recruits and activates different proteins and complexes involved in DNA metabolism
Semaphorin-6D	Q8NFY4	SEMA6D	6.191382	2.630261	Membrane bound-signaling molecule
Platelet factor 4	P02776	PF4	6.035233	2.59341	
Protein S100-A8	P05109	S100A8	5.128781	2.358616	
Lymphocyte-specific protein 1	P33241	LSP1	5.058883	2.338819	
Coatomer subunit beta'	P35606	COPB2	4.978586	2.315736	
Prelamin-A/C	Q5TCI8	LMNA	4.969452	2.313087	Can accelerate smooth muscle cell senescence. It acts to disrupt mitosis and induce DNA damage in vascular smooth muscle cells, leading to mitotic failure, genomic instability, and premature senescence.
NACHT, LRR and PYD domains-containing protein 14	Q86W24	NLRP14	4.761412	2.25139	
Rho GDP-dissociation inhibitor 2	P52566	ARHGDIB	4.694546	2.230986	G-protein modulator signaling molecule Regulator of the cytoskeleton
Nck-associated protein 5	A0A0A0MS79	NCKAP5	4.566907	2.191217	
E3 ubiquitin-protein ligase HECTD1	Q9ULT8	HECTD1	4.552745	2.186737	Directly transfers the ubiquitin to targeted substrates
Glucosidase 2 subunit beta	P14314	PRKCSH	4.532818	2.180408	N-linked glycan-processing enzyme
Platelet factor 4 variant	P10720	PF4V1	4.359601	2.124196	Chemokine
Coactosin-like protein	Q14019	COTL1	4.237172	2.083102	Actin-binding protein
Nascent polypeptide-associated complex subunit alpha, muscle-specific form	E9PAV3	NACA	4.22137	2.077711	Basic helix-loop-helix transcription factor
Axonemal dynein light chain domain-containing protein 1	Q5T1B0	AXDND1	4.047582	2.01706	Axonemal dynein lightchain domain-containing protein 1
Glucose-6-phosphate 1-dehydrogenase	P11413	G6PD	3.925691	1.972947	

Dynein heavy chain 6, axonemal	Q9C0G6	DNAH6	3.783173	1.919597	Microtubule binding motor protein
Serine--tRNA ligase, cytoplasmic	P49591	SARS	3.755766	1.909107	RNA binding protein aminoacyl-tRNA synthetase
Heat shock protein HSP 90-beta	P08238	HSP90AB1	3.698296	1.886861	Chaperone involved in signal transduction, protein folding and degradation and morphological evolution; is thought to play a role in gastric apoptosis and inflammation
Immunoglobulin heavy constant gamma 2	P01859	IGHG2	3.615016	1.854002	
Cytoplasmic protein NCK2	O43639	NCK2	3.576822	1.838678	Involved in cytoskeletal reorganization
Histone H2B type 1-D	P58876	HIST1H2BD	3.565417	1.834071	Component of the nucleosomes which, in addition to wrap and compact DNA into chromatin, play a central role in transcription regulation, DNA repair, DNA replication and chromosome stability
Isoform 2 of Dystonin	Q03001-8	DST	3.406367	1.768234	
Coronin-1A	P31146	CORO1A	3.37597	1.755302	Non-motor actin binding proteins<br>Involved in a variety of cellular processes, including cell cycle progression, signal transduction, apoptosis, and gene regulation
Heterogeneous nuclear ribonucleoprotein Q	O60506	SYNCRIP	3.3363	1.738249	
Isoform 2 of Vasoactive intestinal polypeptide receptor 2	P41587-2	VIPR2	3.229025	1.691099	
Filamin-C	Q14315	FLNC	3.207691	1.681535	
Xin actin-binding repeat-containing protein 2	A4UGR9	XIRP2	3.191186	1.674093	

Lipopolysaccharide-responsive and beige-like anchor protein	P50851	LRBA	3.189025	1.673115	
Hemoglobin subunit gamma-2	P69892	HBG2	3.098604	1.631618	
Histone H2A type 1-H	Q96KK5	HIST1H2AH	3.073629	1.619943	
Hemoglobin subunit delta	P02042	HBD	3.05859	1.612867	
Cofilin-1	P23528	CFL1	2.978019	1.574353	Regulator of the cytoskeleton
Isoform 2 of Heat shock protein HSP 90-alpha	P07900-2	HSP90AA1	2.974058	1.572433	Involved in cell cycle control and signal transduction
HCG1745306, isoform CRA_a	G3V1N2	HBA2	2.895402	1.533764	
Isoform 4 of Xin actin-binding repeat-containing protein 2	A4UGR9-4	XIRP2	2.877506	1.524819	
Transforming protein RhoA	P61586	RHOA	2.815206	1.49324	Associated with cytoskeleton organization. Overexpression of this gene is associated with tumorcell proliferation and metastasis.
Hemoglobin subunit alpha	P69905	HBA1	2.811146	1.491158	Human alpha globin
6-phosphogluconate dehydrogenase, decarboxylating	P52209	PGD	2.77722	1.473642	
Peroxiredoxin-1 (Fragment)	A0A0A0MSI0	PRDX1	2.758974	1.464132	
Synaptotagmin-like protein 2	A0A0U1RR07	SYTL2	2.732614	1.450282	Plays a role in RAB27A-dependent vesicle trafficking and controls melanosome distribution in the cell periphery
Isoform 4 of Platelet glycoprotein 4	P16671-4	CD36	2.718027	1.44256	
Isoform 5 of Serine/threonine-protein kinase tousled-like 1	Q9UKI8-5	TLK1	2.71272	1.43974	Serine/threonine kinase that may be involved in the regulation of chromatin assembly
Phosphoglycerate kinase 1	P00558	PGK1	2.701341	1.433676	Cofactor for polymerase alpha
G patch domain-containing protein 4	A0A0A0MRK1	GPATCH4	2.657459	1.410047	
Versican core protein	P13611	VCAN	2.60464	1.381084	

Actin-related protein 2/3 complex subunit 3	O15145	ARPC3	2.556426	1.354128	
Tyrosine-protein kinase ITK/TSK	Q08881	ITK	2.551291	1.351227	
Ubiquitin-40S ribosomal protein S27a (Fragment)	J3QTR3	RPS27A	2.550911	1.351013	
Isoform 3 of Ubiquitin carboxyl-terminal hydrolase 7	Q93009-3	USP7	2.517224	1.331834	Deubiquitinates target proteins such as p53 between others, promoting proteasomal degradation
Thrombospondin-1	P07996	THBS1	2.483254	1.312232	Adhesive glycoprotein that mediates cell-to-cell and cell-to-matrix interactions.
Hemoglobin subunit beta	P68871	HBB	2.482989	1.312078	Hemoglobin subunit beta
ATP-binding cassette sub-family A member 1	O95477	ABCA1	2.461312	1.299428	
Protein unc-13 homolog D	Q70J99	UNC13D	2.411428	1.269888	Appears to play a role in vesicle maturation during exocytosis and is involved in regulation of cytolytic granules secretion
Calreticulin	P27797	CALR	2.394849	1.259935	Multifunctional protein that acts as a major Ca(2+)-binding (storage) protein
Na(+)/H(+) exchange regulatory cofactor NHE-RF1	O14745	SLC9A3R1	2.366835	1.242959	
Coiled-coil domain-containing protein 39	Q9UFE4	CCDC39	2.360073	1.238831	Motility of cilia and flagella
Prohibitin	P35232	PHB	2.345002	1.229589	Is proposed to play a role in human cellular senescence and tumor suppression. Antiproliferative activity is reported to be localized to the 3' UTR, which is proposed to function as a trans-acting regulatory RNA
Calpastatin	P20810	CAST	2.342036	1.227763	

		**DOWN**			
Cytosolic carboxypeptidase 2	Q5U5Z8	AGBL2	0.402469	-1.31305	G-protein metalloprotease
Cysteine and glycine-rich protein 1	P21291	CSRP1	0.400022	-1.32185	
Thrombospondin-2	P35442	THBS2	0.399148	-1.32501	
Serotransferrin	P02787	TF	0.386377	-1.37192	Receptor serine protease transfer/carrier protein; transports iron from the intestine, reticuloendothelial system, and liver parenchymal cells to all proliferating cells in the body
Kinesin-like protein KIF18A	Q8NI77	KIF18A	0.36719	-1.4454	Microtubule binding motor protein; kinesin
Isoform 3 of Polycystic kidney disease 2-like 1 protein	Q9P0L9-3	PKD2L1	0.360671	-1.47124	
Kinesin-like protein KIF22	Q14807	KIF22	0.346076	-1.53084	Involved in spindle formation and the movements of chromosomes during mitosis and meiosis.
Rho-associated protein kinase 1	Q13464	ROCK1	0.343188	-1.54293	
Isoform PKP3b of Plakophilin-3	Q9Y446-2	PKP3	0.34304	-1.54355	Participates in linking cadherins to intermediate filaments in the cytoskeleton
2,4-dienoyl-CoA reductase, mitochondrial	Q16698	DECR1	0.342193	-1.54712	
Myosin regulatory light chain 12A	P19105	MYL12A	0.341905	-1.54833	Involved in DNA damage repair by sequestering the transcriptional regulator apoptosis-antagonizing transcription factor (AATF)/Che-1 which functions as a repressor of p53-driven apoptosis
Xaa-Pro dipeptidase	P12955	PEPD	0.333076	-1.58608	Metalloprotease; nucleic acid binding transcription factor; recycling of proline

Protein FAM47A	A0A0C4DGW7	FAM47A	0.326965	-1.61279	
Fructose-bisphosphate aldolase C	P09972	ALDOC	0.297765	-1.74776	
Crk-like protein	P46109	CRKL	0.290316	-1.78431	
ERC protein 2 (Fragment)	H7C4G9	ERC2	0.28808	-1.79546	G-protein modulator; membrane traffic protein; regulator of neurotransmitter release
Zinc finger protein 492	Q9P255	ZNF492	0.282596	-1.82318	
Band 3 anion transport protein	P02730	SLC4A1	0.262393	-1.9302	Transporter; anion exchanger in the erythrocyte plasma membrane
PDZ and LIM domain protein 7	Q9NR12	PDLIM7	0.254756	-1.97281	
Eukaryotic translation initiation factor 4 gamma 2	P78344	EIF4G2	0.244238	-2.03364	Plays a role in the switch from cap-dependent to IRES-mediated translation during mitosis, apoptosis and viral infection. Cleaved by some caspases and viral proteases.
Ankyrin-3	Q12955	ANK3	0.232868	-2.10242	Links the integral membrane proteins to the underlying spectrin-actin cytoskeleton and plays key roles in activities such as cell motility, activation, proliferation, contact, and the maintenance of specialized membrane domains.
Unconventional myosin-XVIIIa	Q92614	MYO18A	0.220753	-2.17949	
Citrate synthase, mitochondrial (Fragment)	H0YH82	CS	0.208337	-2.26301	Krebs tricarboxylic acid cycle enzyme
Integrin alpha-M	P11215	ITGAM	0.150349	-2.73361	Integral membrane protein
UTP--glucose-1-phosphate uridylyltransferase	Q16851	UGP2	0.144282	-2.79304	
Guanine nucleotide-binding protein G(z) subunit alpha	P19086	GNAZ	0.142569	-2.81026	
Isoform 2 of Protocadherin gamma-B6	Q9Y5F9-2	PCDHGB6	0.140918	-2.82707	

D-aminoacyl-tRNA deacylase 1	Q8TEA8	DTD1	0.136309	-2.87505	
Ras GTPase-activating protein 3	Q14644	RASA3	0.129194	-2.95239	
Isoform 2 of Pleckstrin homology domain-containing family O member 2	Q8TD55-2	PLEKHO2	0.115905	-3.10899	Part of the Innate immune system
Cardiomyopathy-associated protein 5	Q8N3K9	CMYA5	0.099686	-3.32646	
Myosin-11	P35749	MYH11	0.085022	-3.55602	
Spectrin beta chain, erythrocytic	P11277	SPTB	0.048564	-4.36397	Cell membrane organization and stability
Transcription factor E2F7	Q96AV8	E2F7	0.039747	-4.65302	Regulation of cell cycle progression
