# Supplementary material for: Differential Expression of Proteins in an Atypical Presentation of Autoimmune Lymphoproliferative Syndrome
Source: Int J Mol Sci. 2022 May 11;23(10):5366. doi: 10.3390/ijms23105366 (PMC9140392; doi:10.3390/ijms23105366)
Supplement: Supplementary file 1 [file ijms-23-05366-s001.zip › ijms-1692249-supplementary/Supplementary Material/Supplementary Table S2.pdf]

Supplementary Table S2. Exclusively expressed proteins in control and ALPS condition. Proteins detected with CV ≤ 0.15, two peptides per protein (one unique) and ANOVA $p \leq 0.05$.

CONTROL			
Protein	Accession	Gene	Function
Serine/threonine-protein kinase	Q8TDC3	BRSK1	Serine/threonine-protein kinase that plays a key role in polarization of neurons and centrosome duplication.
Calcineurin B homologous protein 2	O43745	CHP2	Regulates cell pH
Hippocalcin-like protein 1	P37235	HPCAL1	May be involved in the calcium-dependent regulation of rhodopsin phosphorylation and may be of relevance for neuronal signaling in the central nervous system
Coiled-coil domain-containing protein 138	Q96M89	CCDC138	Coiled-coil domain-containing protein 138
AMSH-like protease	Q96FJ0	STAMBPL1	Metallopeptidase
Mitochondrial ornithine transporter 2	Q9BXI2	SLC25A2	
Single-stranded DNA-binding protein, mitochondrial (Fragment)	C9K0U8	SSBP1	Involved in mitochondrial DNA replication.
Bifunctional purine biosynthesis protein PURH	P31939	ATIC	
Copine-7	Q9UBL6	CPNE7	Membrane trafficking
Immunoglobulin heavy constant alpha 1	P01876	IGHA1	
Dedicator of cytokinesis protein 1	Q14185	DOCK1	Dedicator of cytokinesis 1; phagocytosis and cell migration
Isoform 2 of Dihydrolipoyl dehydrogenase, mitochondrial	P09622-2	DLD	
CD48 antigen	A0A087X1S7	CD48	
Isoform 2 of Tectonic-3	Q6NUS6-2	TCTN3	Is involved in apoptosis regulation
Protein SDA1 homolog	E7EW05	SDAD1	
PWWP domain-containing protein MUM1	Q2TAK8	MUM1	
PDZ and LIM domain protein 5	Q96HC4	PDLIM5	
Alpha-ketoglutarate-dependent dioxygenase FTO	A0A1B0GTC3	FTO	
Transcription initiation factor TFIID subunit 13	Q15543	TAF13	Transcription factor
Double zinc ribbon and ankyrin repeat-containing protein 1	A0A0A0MRE2	DZANK1	
Zinc finger protein 385A (Fragment)	H0YI55	ZNF385A	RNA-binding protein that affects the localization and the translation of a subset of mRNA.
Potassium voltage-gated channel subfamily H member 7	A0A087WZQ1	KCNH7	
Isoform 2 of PCI domain-containing protein 2	Q5JVF3-2	PCID2	Component of the TREX-2 complex (transcription and export complex 2), which regulates mRNA export from the nucleus
cTAGE family member 9	A4FU28	CTAGE9	Growth factor

Calcium-dependent secretion activator 2	A0A087X1P3	CADPS2	
Phosphomevalonate kinase	Q15126	PMVK	Isoprenoid biosynthesis
CST complex subunit STN1	Q9H668	STN1	Subunit of an alpha accessory factor that stimulates the activity of DNA polymerase-alpha-primase, the enzyme that initiates DNA replication
Neurolysin, mitochondrial	E9PCB6	NLN	Metallopeptidase
HEAT repeat-containing protein 4	Q86WZ0	HEATR4	
Isoform 2 of Enhancer of polycomb homolog 1	Q9H2F5-2	EPC1	Chromatin binding protein; linked to apoptosis, DNA repair, skeletal muscle differentiation, gene silencing, and adult T-cell leukemia/lymphoma
Pumilio homolog 1	Q14671	PUM1	Translation factor; involved in translational regulation of embryogenesis, and cell development and differentiation
FYVE, RhoGEF and PH domain-containing protein 6	F8VY01	FGD6	
Endothelin-converting enzyme 2	O60344	ECE2	
Isoform 2 of Electrogenic sodium bicarbonate cotransporter 4	Q9BY07-2	SLC4A5	
Probable ATP-dependent RNA helicase DDX46	Q7L014	DDX46	
**ALPS**			
Myotrophin	P58546	MTPN	
HLA class I histocompatibility antigen, A-68 alpha chain	P01891	HLA-A	
Activated RNA polymerase II transcriptional coactivator p15	P53999	SUB1	
Serine/threonine-protein kinase SMG1	Q96Q15	SMG1	Involved in both mRNA surveillance and genotoxic stress response pathways; required for optimal p53/TP53 activation after cellular exposure to genotoxic stress
Lysine-specific demethylase 6B	O15054	KDM6B	Transcripton factor; demethylase control of chromatin organization and gene silencing
Protein tyrosine phosphatase domain-containing protein 1	A2A3K4	PTPDC1	Phosphatidylinositol-transfer protein beta isoform
Phosphatidylinositol transfer protein beta isoform	P48739	PITPNB	
Ran-specific GTPase-activating protein (Fragment)	C9JIC6	RANBP1	
Heterogeneous nuclear ribonucleoprotein A1	P09651	HNRNPA1	
Laminin subunit gamma-1	P11047	LAMC1	
Isoform 3 of DNA repair protein complementing XP-G cells	P28715-3	ERCC5	DNA repair protein. Single-strand specific DNA endonuclease makes the 3′ incision in DNA excision repair following UV-induced damage and works too in other cellular processes, including RNA polymerase II transcription, and transcription-coupled DNA repair

Patatin-like phospholipase domain-containing protein 7	Q6ZV29	PNPLA7	
A-kinase anchor protein SPHKAP	Q2M3C7	SPHKAP	
Small RNA 2'-O-methyltransferase (Fragment)	X6RGV9;	HENMT1	
SLIT-ROBO Rho GTPase-activating protein 3	O43295	SRGAP3	G-protein modulator
Galectin-1	P09382	LGALS1	Cell adhesion molecule. Signaling molecule Galectin that can act in the regulation of cell proliferation
